# Selected abstracts from the 24th Annual Meeting of the Society in Europe for the Simulation Applied to Medicine: Bilbao, Spain. 27-29 June 2018

**DOI:** 10.1186/s41077-018-0066-5

**Published:** 2018-06-27

## R1 Simulation training in stroke thrombolysis: reducing door to needle times to less than 15 minutes

### Soffien Chadli Ajmi^1,2,3^, Rajiv Advani^4,5^, Lars Fjetland^6,7^, Kathinka D. Kurz^6,7^, Thomas Lindner^8^, Sigrun Qvindesland^9^, Hege Ersdal^3,8^, Martin W. Kurz^1,2,10^

#### ^1^Department of Neurology, Stavanger University Hospital, Stavanger, Norway; ^2^Neuroscience Research Group, Stavanger University Hospital, Stavanger, Norway; ^3^Faculty of Health Sciences, University of Stavanger, Stavanger, Norway; ^4^Department of Neurology, Østfold Hospital, Kalnes, Norway; ^5^University Hospital and Faculty of Medicine, University of Bergen, Bergen, Norway; ^6^Department of Radiology, Stavanger University Hospital, Stavanger, Norway; ^7^Radiological Research Group, Stavanger University Hospital, Stavanger, Norway; ^8^Department of Anesthesiology and Intensive Care, Stavanger University Hospital, Stavanger, Norway; ^9^Stavanger Acute Medicine Foundation for Education and Research, Stavanger, Norway; ^10^Faculty of Medicine, University of Bergen, Bergen, Norway

##### **Correspondence:** Soffien Chadli Ajmi (soffiena@yahoo.com)

**Ethics statement**

The authors declare that they have followed the guidelines for scientific integrity and professional ethics. The article does not contain any studies with human or animal subjects.

**Introduction & Aims**

Stroke is one of the leading causes of morbidity and mortality worldwide. In eligible patients with acute ischemic stroke, early treatment with intravenous thrombolysis is crucial for a good patient outcome. We introduced simulation training sessions in conjunction with an improved treatment protocol as part of a quality improvement project to reduce door-to-needle times in stroke thrombolysis.

**Methods**

A questionnaire assessing our preexisting treatment protocol was sent to all members of the stroke team. A panel of experts reviewed the responses and suggested potential changes to streamline the treatment protocol. In February 2017, we introduced the new protocol along with weekly videotaped in-situ scenario based simulation sessions with all stroke team members as participants. Previous stroke patients acted as markers. Kirkpatrick’s four-level training evaluation model was used for assessment. Here we present 1) Participant reactions (level 1) on a Likert item from 0-10, and 2) Median door-to-needle times in stroke thrombolysis, a measure of clinical behavioral change (level 3), using a statistical process control method. Simulated performance and long term patient outcomes will be assessed in future analysis.

**Results & Discussion**

Participant reactions were predominantly positive. Self-perceived learning scored a median of 8 (IQR 7-9). We compared door-to-needle times for 478 prospectively included patients with acute ischemic stroke treated with intravenous thrombolysis at our hospital from January 2014 – July 2017. There was a significant reduction in median door-to-needle time from 27 (IQR 19-41) to 13 minutes (IQR 9-21, *p*<0.001) for the 78 patients in the post-intervention group. The results remained significant regardless of time of admission. There were no significant changes in the rate of stroke mimics, prehospital time or fatal intracranial hemorrhage.

Simulation training in conjunction with protocol improvement led to an immediate and significant reduction of median door-to-needle time in stroke thrombolysis (Fig. 1). To our knowledge, no other published data have shown lower median treatment times. Combining simulation training with protocol change holds promise as a method both for effective implementation and significant results in attempts to reduce in-hospital delays in stroke thrombolysis. Effects on non-technical skills, provider variability and long term patient outcomes are yet to be evaluated.

**Fig. 1 (abstract R1).**
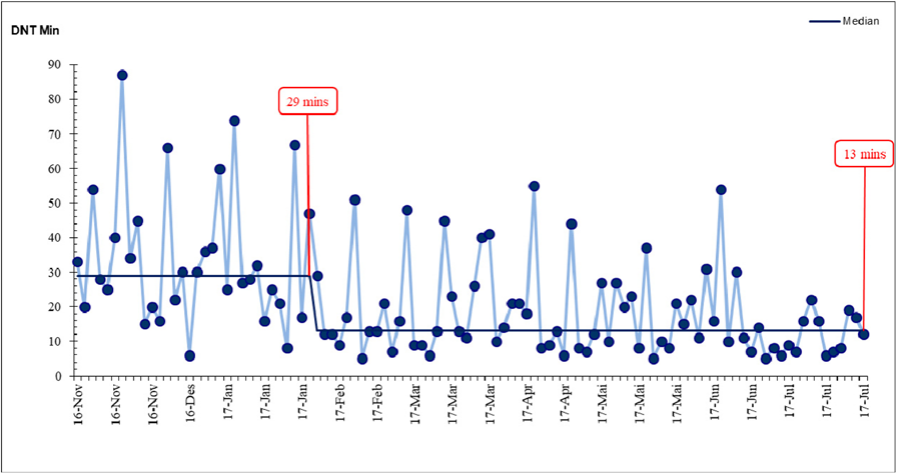
Individual door-to-needle times with group medians before and after quality improvement

## R2 Sequential Simulation (SqS): an empirical and theoretical model

### Sharon Marie Weldon^1,2^ (s.m.weldon@gre.ac.uk)

#### ^1^Faculty of Education & Health, University of Greenwich, London, UK; ^2^Barts Health NHS Trust, London, UK

**Ethics statement**

The authors declare that all procedures followed were in accordance with the ethical standards of the responsible committee on human experimentation (institutional and national ) and with the Helsinki Declaration of 1975 ( In its most recently amended version ). Informed consent was obtained from all patients/participants included in the study. All institutional and national guidelines for the care and use of laboratory animals were followed.

**Introduction & Aims**

Sequential Simulation (SqS) is the physical re-enactment of healthcare-related scenarios. Its main feature is that it recreates connected components of care in place of single episodes. It, therefore, represents the healthcare system from the perspective of those who journey through it (the patients and their carers) and those who populate it (healthcare professionals and other employees), aiming to bridge gaps and discrepancies that often only they experience.

Due to the various care pathways that a patient could journey through, this form of simulation is complex to design and has not often been utilised. This paper, therefore, aims to explore its structure and potential, to provide clinical educators with a model to aid in the design process, and a better understanding of its effectiveness and affordances.

**Methods**

Existing simulation and other sector theories were combined with empirical data of SqS simulations to identify the key components of a successful design. The design concepts (key components of the simulation design) identified through a literature framework and systematic coding of the various collected process and observational data, were tested and refined through a modified Delphi-technique tool that research subjects used and completed while designing successful (e.g. objectives achieved) SqS Simulations. Comparative analysis of the different applications of the model, narrative case studies of the model in use, and the presentation of a conceptual and process model bring together the studies various iterations, as well as concluding the research through the presentation of the model.

**Results & Discussion**

The key outcomes of this research are the identification of the key components that constitute SqS simulations and its affordances; the different considerations required for the different simulation objectives; the presentation of three validated and refined conceptual and process models that can be used in practice to design SqS simulations.

The unique affordances of the model developed are that it is grounded in existing multi-sector knowledge (theoretical data); it is grounded in detailed observations of actual simulation practice (empirical data); it embraces the complexities of the design and enables a structured design process; it provides an adaptable and documented framework; it is validated and tested in practice by clinicians; and it provides key design features and differences between the required objectives.

Therefore, this research has outlined the structure and affordances of a sophisticated approach to simulation design that should allow others to further test and refine the SqS concept.

## R3 Debriefing with team deliberate practice: an instructional design to enhance the performance of undergraduate nursing students in recognising the deteriorating patient

### Alan Platt^1^, Peter McMeekin^1^, Linda Prescott-Clements

#### ^1^Northumbria University, Newcastle upon Tyne, UK; ^2^NHS Litigation Authority, London, UK

##### **Correspondence:** Alan Platt (alan.platt@northumbria.ac.uk)

**Ethics statement**

The authors declare that they have followed the guidelines for scientific integrity and professional ethics. The article does not contain any studies with human or animal subjects.

**Introduction & Aims**

The purpose of this paper is to give an overview of the development of debriefing with team deliberate practice (DwTDP) as an innovative instructional design. The author will present their research findings and discuss its application to healthcare education.

A key competency that undergraduate nurses have to achieve is that of the early recognition of clinical deterioration of patients the Nursing and Midwifery Council1, and as advocated by the Chief Medical Officer2 simulation-based education is an important methodology in achieving this. Despite a growing evidence base for the use of simulation as a learning and teaching methodology Anderson and colleagues3 found a wide variation in the quality of delivery and recommended further research into those instructional design features that enhance learning. To meet this challenge, the author developed an innovative simulation-based educational enhancement entitled DwTDP. This was based on the deliberate practice framework4 and incorporated key elements from team working and debriefing theory.

**Methods**

Using a quasi-experimental longitudinal pre-post design, the researcher explored the effect of DwTDP on the performance of second year adult nursing students (N = 93) over a one-year period. Naturally occurring student groups were randomised into either the intervention arm (n = 8), who received DwTDP, or the comparison arm (n = 8) who received a traditional SBE.

**Results & Discussion**

Pre and post video data of the students’ performance was captured and collected at three time points over the course of the year. This was analyzed using a series of statistical techniques. An Independent t-test found that there was no statistically significant effect on the participant’s performance during the individual phases. However, a mixed ANOVA analysis identified that over time the DwTDP intervention led to a statistically significant improvement in the performance of the participants (F(1, 6) = 19.12, p = .005).

In phase 1 the participant’s time on task from the intervention group also showed statistical improvement (t(14) = 5.12, *p*<.001), with a very large effect size (d = 2.56). Although the effect sizes remained large in the other two phases the Independent t-tests were not statistically significant.

The results inferred that the DwTDP intervention was a feasible approach to use within nurse education. It enhanced the participant’s performance in recognising a deteriorating patient overtime and initially improved their response times. The author therefore recommends the use of this approach within adult nursing pre-registration curricula and further research into its efficacy with other healthcare professionals.

## R4 Behavioral impact on communication during routine patient care in a pediatric intensive care unit following simulation training

### Francis Ulmer^1^, Andrea Lutz^2^, Fabienne Mueller^1^, Fabian Buergi^2^, Robert Greif^2^

#### ^1^Department of Pediatrics, Bern University Hospital, University of Bern, Bern, Switzerland; ^2^Department of Anesthesiology and Pain Therapy, Bern University Hospital, University of Bern, Bern, Switzerland

##### **Correspondence:** Francis Ulmer (fulmer52@gmail.com)

**Ethics statement**

We, the authors declare that all procedures followed were in accordance with the ethical standards of the responsible committee on human experimentation (institutional and national) and with the Helsinki Declaration of 1975 (in its most recently amended version). Informed consent was obtained from all patients/participants included in the study. All institutional and national guidelines were followed.

**Introduction & Aims**

Effective communication helps minimize medical errors leading to improved team-performance among healthcare professionals when treating critically ill patients. Closed loop communication (CLC) is routinely applied in high-risk industries but remains underutilized in healthcare. CLC prevalence in pediatric intensive care units (PICU) remains unknown. Interprofessional and multidisciplinary in-situ simulation serves as an educational tool to introduce, practice and appreciate the efficacy of CLC. This observational before and after study investigates behavioral changes in communication in the clinical setting brought on by high-fidelity simulation.

**Methods**

Following ethics approval and informed consent, the communication patterns of PICU nurses, who had no prior simulation experience, were observed during routine clinical bed-side patient care. One month before (baseline), as well as 1 and 3 months after simulation, 2 trained raters independently recorded nurse communication relative to call-out (CO), uttered by the sender, and check-back (ChB), uttered by the recipient (Fig. 1). The impact of simulation on communication patterns was analyzed quantitatively; sender and recipient communications were analyzed separately. Wilcoxon signed-rank test and Friedman test compared observed changes in communication; data are presented as median an interquartile range.

**Results & Discussion**

Results

Fifteen PICU nurses were included. At baseline the ratio of direct to nondirected call outs was 17:1 (11:1–35:1) for senders and 18:1 (10:1–38:1) for recipients. A significant decrease in no-ChB communication was observed when study participants acted as both recipients (*p*=0.001) and senders (*p*=0.005). In the remaining ChB-categories nonverbal ChBs decreased, verbal ChBs and call-backs increased without reaching statistical significance. Among senders, verbal-ChBs increased significantly when preceded by a directed command (*p*=0.021) or directed information (*p*=0.005), as did call-backs that were preceded by a directed question (*p*=0.012). The Inter-Rater Reliability was 0.51 (0.35-0.63) (Spearman’s correlation coefficient).

Discussion

To our knowledge this is the first study describing prevalence and pattern of communication among PICU nurses during routine patient care. Simulation naïve PICU nurses are more likely to employ verbal ChBs in response to directed information and directed commands, more likely to answer using call backs in response to directed questions and less likely to respond with nonverbal ChBs as a result of simulation training. These findings suggest that simulation training alters staff communication behaviors in the clinical setting by discouraging communication patterns that are prone to lead to misunderstandings and by promoting communication patterns that strengthen the unaltered flow of information in the intensive care unit, which is likely to enhance patient safety.

**Fig. 1 (abstract R4).**
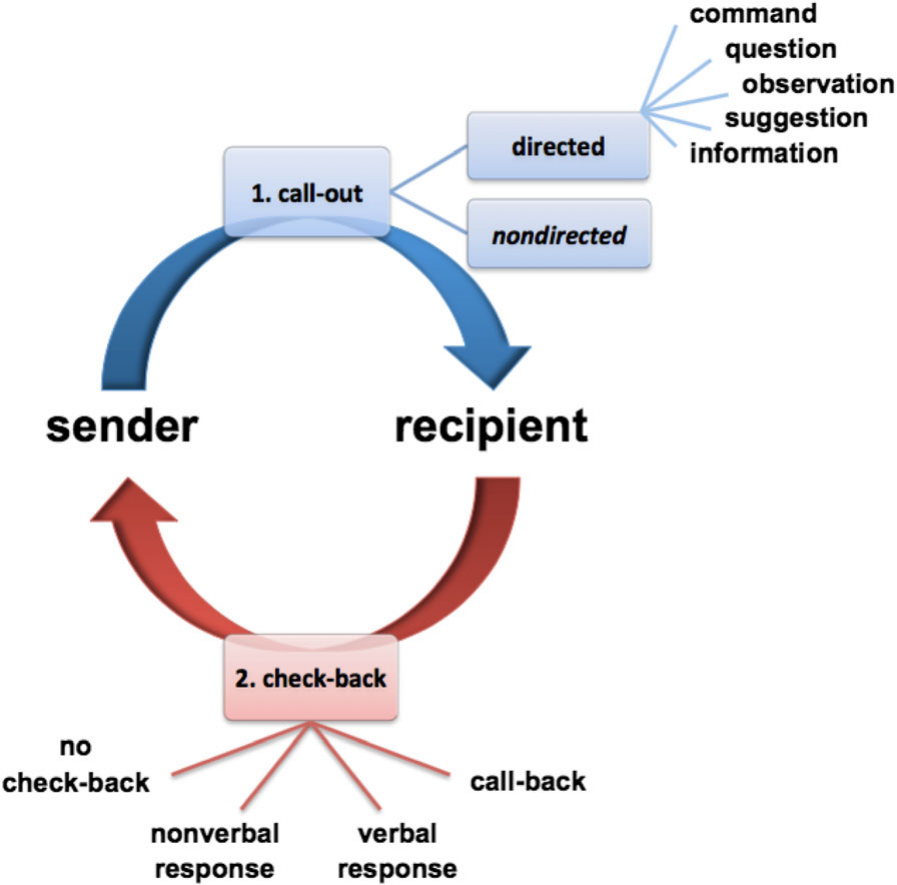
See text for description

## R5 Comparative upper limb motion study of surgeons during a simulated laparoscopic test

### María Gloria Álvarez^1^, Horacio Pace^2^, Andrés Conejero^2^, Jose Dolz^1^

#### ^1^Área de Simulación Clínica y Seguridad del Paciente, Hospital Universitari i Politècnic La Fe, Valencia, Spain; ^2^Instituto de Diseño y Fabricación, Universitat Politècnica de Valencia, Valencia, Spain

##### **Correspondence:** Jose Dolz (dolz_jos@gva.es)

**Ethics statement**

The authors declare that they have followed the guidelines for scientific integrity and professional ethics. Informed consent was obtained from all participants included in the study.

**Introduction & Aims**

Laparoscopic technique has demonstrated numerous advantages compared to open conventional surgery. Nevertheless, this procedure increases the surgeon fatigue and thus the potential to commit errors that may harm the patient during the operation. The post-surgery pain also increases as the configuration of the instruments force the surgeons to adopt non-neutral postures during the practice. “Postural Freedom” is defined as the capacity of the surgeons to adapt their position and movements in front of the operation table to reach comfort. This study reveals how this could help surgeons to improve the postural hygiene and how previous experience in laparoscopic surgery may influence the results.

**Methods**

14 subjects participated in the study, 6 of them with no previous experience in laparoscopic surgery. They performed a surgical simulation with two instrument configurations, a conventional pistol grip instrument and a prototype with an articulated element. The articulated prototype allowed the subjects to adopt more comfortable postures (neutral positions). During the test, the subjects were recorded to analyze their motions with an adaptation of Rapid Entire Body Assessment (REBA) method. In order to evaluate the positions adopted for the surgeon, a motion capture set tracked their movements during a circuit traced inside a box-trainer.

**Results & Discussion**

This study showed a significant improvement in the number of neutral positions adopted by subjects working with the articulated prototype. More specifically, these improvements were 34,88% higher in arm abduction-adduction, 30,56% higher in forearm flexion and 20,99% higher in arm flexion-extension. Collected data also showed significant differences when the subjects had previous experience in Minimally Invasive Surgery (MIS) procedures. The fact that these subjects adopted worse postures with the articulate prototype would probably be due to the influence of the experience acquired with conventional instruments. The current study demonstrates that, when “Postural Freedom” is improved, surgeons achieve greater percentages of neutral positions. In addition, we have seen that the implementation of an articulated element between handle and stylus could be a key element to avoid risk postures during laparoscopic surgery.

## R6 The perceived teamwork effectiveness, occupational self-efficacy and work-related stress after the multidisciplinary medical staff teamwork in-situ simulation training

### Kärt Pielberg (kart.pielberg@gmail.com)

#### Pelgulinna Simulation Centre and West Tallinn Central Hospital, Women’s Clinic and Tallinn University, Tallinn, Estonia

**Ethics statement**

The authors declare that they have followed the guidelines for scientific integrity and professional ethics. The article does not contain any studies with human or animal subjects.

**Introduction & Aims**

Pelgulinna Simulation Center, created in 2015, provides in-situ simulation trainings for obstetric and neonatology teams. The goal is to practice technical skills and gain knowledge as a team providing an effective medical service. To analyze the effectiveness a questionnaire-based study was conducted in the framework of Organizational Behavior Master’s program at Tallinn University, Estonia. The specific aim of this study was to explore the changes in teamwork effectiveness, occupational self-efficacy and work-related stress after the multidisciplinary teamwork training.

**Methods**

The survey was performed during the period of January-April, 2017. In conjunction with a total of 11 interactive in-situ simulation trainings, taking place at different Estonian hospitals, 100 specialists were submitted to a questionnaire measuring work-related stress, occupational self-efficacy and teamwork effectiveness. An identical anonymous (coded) questionnaire was filled out by participants before, as well as three weeks after the training. Statistical analysis (mean equivalence, T-test, Pearson correlation coefficient and Cronbach’s alpha) was performed using the IBM SPSS Statistic 24.0 program, considering a confidence level of *p*<0.05 as significant.

**Results & Discussion**

Among the 69% of attendees answering to the questionnaire, several occupational groups were represented (largest groups being midwives (45%) and gynaecologists (19%)). On average, the staff had been actively practicing on their field for 15 years (M=12,0; SD=12,99), whereas 71% of them had a previous simulation training experience. The study revealed that healthcare workers perceived the teamwork as more efficient, and the team coordination, cooperation and exchange of information as improved after the training (*p*=0,032). It was evident that employees perceived an improvement in the team’s performance, measured by significant changes in self-efficacy before and after the training (*p*=0,01). A clear increased job satisfaction (*p*<0,05) was revealed, whereas there were no statistical differences detected between the levels of perceived work stress before and after the training (*p*=0,24). Trainings had no effect on the responsibility pressure (*p*=0,22), as well as on the conflict of roles (*p*=0,81). No differences were found between individuals with or without previous experience of simulations, neither between representatives of various disciplines.

In conclusion, the results of the study showed the effectiveness of simulation-based training by improving the perceived teamwork effectiveness in terms of collaboration, interaction and work environment adaptation. Also, a positive change in perceived work-related self-efficacy, job performance and satisfaction among employees was observed. The study showed the need for such simulations for medical staff and for the development of relevant statistical models measuring the training outcomes.

## R7 Development of a venipuncture simulator manikin for health education

### Geana Silva dos Santos, Marcia Elisa Soares Echeveste, Érico Marcon

#### Federal University of Rio Grande do Sul, Farroupilha, Brasil

##### **Correspondence:** Geana Silva dos Santos (geana.santos@ufrgs.br)

**Ethics statement**

The authors declare that they have followed the guidelines for scientific integrity and professional ethics. The article does not contain any studies with human or animal subjects.

**Introduction & Aims**

Simulation has been adopted as the standard in highly complex and technical professions, where there is a high risk to human life, such as, for example, aerospace, military, healthcare and other professions. In general, simulated practices in teaching laboratories use manikins, anatomical models and equipment similar to those of hospital units, creating the most realistic environment possible. In order to contribute to this issue, we aimed to answer the following research question: How can healthcare educators practice the procedure of parenteral injection and venipuncture in the most realistic way possible?. Thus, the purpose of this paper was to describe the beginning of the process of creating a new arm simulator manikin for venipuncture from the requirements survey regarding educational, commercial and technological aspects.

**Methods**

We searched scientific databases filtering for years, from 2003 to 2013, for literature review, where 225 results returned and 26 papers were selected, according to inclusion and exclusion criteria. World patent banks (USA, China, South Korea, etc.) were also surveyed in the period from 1952 to 2013, returning 13 patent registrations, the proportion being the United States (54%), followed by China (31%) and Korea (15%). Finally, we carried out a research with manufacturers of healthcare simulators cited in the literature review, patents and Internet searches. Of the 20 companies listed, 11 manufactured venipuncture arms (Laerdal®, Limbs and Things®, Koken®, etc.), whose product characteristics were analyzed.

**Results & Discussion**

The information was analyzed and summarized in a table of requirements, presenting the development of a venipuncture simulator from educational, commercial and technological aspects. Five categories were described according to (i) functionality, (ii) hybrid simulation, (iii) provision of skills, (iv) ethical aspects and (v) realistic aspects, considered as primary requirements. The definition of requirements is an important step in the development of new products to promote innovation, since they establish an agreement between the customer and the team that develops the product. The requirements are used as a reference for the final validation of the product and they reduce development costs since they avoid rework for not understanding the consumer’s needs (ÁVILA; SPÍNOLA, 2015). The main result of this research was the product requirements table as a contribution to the Industrial Engineering, especially for the process of development of new simulators for the healthcare education area. The evidence found supported the development of an arm simulator manikin for venipuncture with innovative features as shown in the first draft depicted in Fig. 1.

**Fig. 1 (abstract R7).**
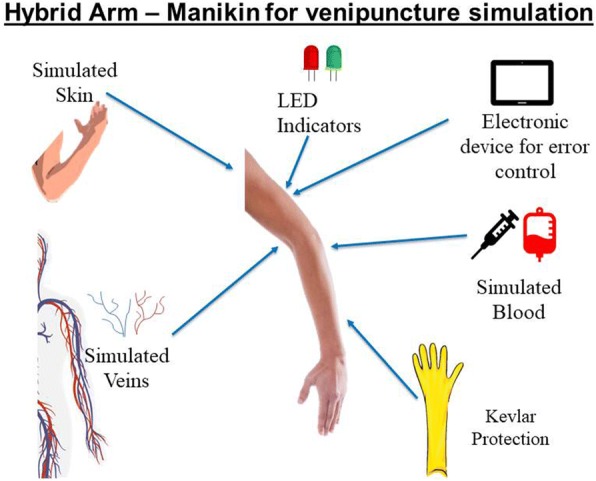
See text for description.

## R8 Assessment of the Wong-Baker Pain Rating Scale inter-rater reliability applied to adult standardised patients by paramedics in a multicultural context

### Padarath Gangaram^1^, Guillaume Alinier^1,2^, Enrico Dippenaar^3^

#### ^1^Hamad Medical Corporation Ambulance Service, Doha, Qatar; ^2^University of Hertfordshire, Hatfield, UK; ^3^Anglia Ruskin University, Chelmsford, UK

##### **Correspondence:** Guillaume Alinier (g.alinier@herts.ac.uk)

**Ethics statement**

The authors declare that all procedures followed were in accordance with the ethical standards of the responsible committee on human experimentation and that informed consent was obtained from all participants included in the study.

**Introduction & Aims**

The appropriate assessment of pain has been identified as a Key Performance Indicator in our Ambulance Service. Currently, patients’ pain level is assessed by our paramedics using the Wong-Baker FACES® Pain Rating Scale as it has been adopted throughout the entire governmental hospital and pre-hospital system in Qatar. Recent findings indicated that the assessment of patients presenting with acute pain by our paramedics is sometimes sub-optimal so we aimed to test this in a controlled and simulated environment.

**Methods**

This prospective, quantitative pilot study was reviewed by the institution’s review board and classified as an improvement study. Five members of staff from the Ambulance Service training department were given a script and prepared as adult standardised patients presenting with differing levels of pain. The study recruited volunteer operational paramedics while they were on standby at various ambulance stations throughout the country. Each participant was exposed to the same five standardised adult patients and the same range of facial expressions in a randomized order. Data was gathered using survey questionnaires to assess inter-rater reliability of their pain assessment.

**Results & Discussion**

35 consenting paramedics were recruited to the study and indicated the pain intensity score of the five standardised adult patients using the Wong-Baker FACES® Pain Rating Scale. This provided a total of 175 pain assessment scores. Overall the inter-rater reliability determined using Fleiss Kappa indicated only a poor to slight agreement of the allocated pain scores, with a wide grouping of responses around the reference pain score levels set for each standardised patient. Most of the allocations were 1 to 2 pain score levels away from the reference standard, although not in a normal distribution with some of the higher reference pain levels receiving lower scores, and vice versa. Overall the sensitivity was poor to very poor throughout.

This study showed that the inter-rater reliability of the participant sample when applying the Wong-Baker FACES® Pain Rating Scale to determine the pain levels of five adult standardised patient cases was poor which could lead to suboptimal pain management of patients. The scoring discrepancy could be attributed to various factors including the multinational population with cultural biases, language barriers, lack of familiarization with the Wong-Baker FACES® Pain Rating Scale, and other environmental factors. Further studies with a larger sample population should be conducted to make the results generalizable and to identify is staff retraining on pain assessment is required.

## R9 When to introduce three-dimensional visualization technology into surgical residency: a randomized controlled trial

### Junyi Gao, Chen Lin, Hua Zheng, Jun Zhao, Hua Yang, Yue Zheng, Yihan Cao, Yufei Chen, Guoliang Wu, Guole Lin, Jianchun Yu, Hanzhong Li, Hui Pan, Quan Liao, Yupei Zhao

#### Peking Union Medical College Hospital, Chinese Academy of Medical Sciences & Peking Union Medical College, Beijing, China

##### **Correspondence:** Junyi Gao (gaojunyipumc@163.com)

**Ethics statement**

The authors declare that all procedures followed were in accordance with the ethical standards of the responsible committee on human experimentation (institutional and national ) and with the Helsinki Declaration of 1975 ( In its most recently amended version ). Informed consent was obtained from all patients/participants included in the study.

**Introduction & Aims**

Three-dimensional (3D) reconstructed images have been increasingly applied for medical education. Although many studies have described the benefits of such applications, the best time to introduce 3D technology into surgical training has not been determined. Therefore, we conducted a randomized study to determine a suitable period for the introduction of this technology.

**Methods**

Seventy-one surgical residents were randomized into 2 groups (two-dimensional computed tomography (CT) group and 3D image group), and they completed a test on anatomy and imaging as well as a questionnaire.

**Results & Discussion**

Results: Post-graduate year 1 (PGY1) residents in the 3D group performed significantly better than those in the CT group, although the third-year residents did not present significant differences in either the score or the time spent answering the questions. Although residents in different years of training held different attitudes toward the difficulty of anatomy and imaging learning, they all showed a high level of acceptance of the 3D training.

Discussion: Although the 3D technology applied in medical education has achieved initial success, few studies have explored the appropriate introduction time of 3D technology to residency programs; thus, we designed this study to explore the optimal timing for introducing 3D technology into a surgical residency program.

As shown in Fig. 1, in the traditional learning model, which could be summarized as “2D → 3D → 2D” , trainees must imagine 3D structures with 2D cross-sectional images. This process is long and difficult and often results in the memorization of inaccurate or even incorrect 3D structures because of the lack of an ability to immediately correct these representations. Our findings suggest that 3D images might be superior in assisting junior residents in establishing 3D models. This new learning model combines 3D and 2D images and might accelerate the learning process by improving the accuracy of 3D structures and deepening the memory of anatomy. We summarize it as “2D + 3D → 3D → 2D”.

Although anatomy and imaging did not appear to be difficult for senior residents, most of them agreed that it could strengthen their systematic training. This result revealed the importance of anatomy and imaging for surgeons and indicated that a gap occurs between the urgent demand for training and the current insufficiency in training.

Conclusion: This study revealed that 3D images improved the junior residents’ performance in imaging reasoning. Thus, systematically introducing 3D images early in a surgical resident training program may help produce a better anatomy-imaging-surgery system.

**Fig. 1 (abstract R9).**
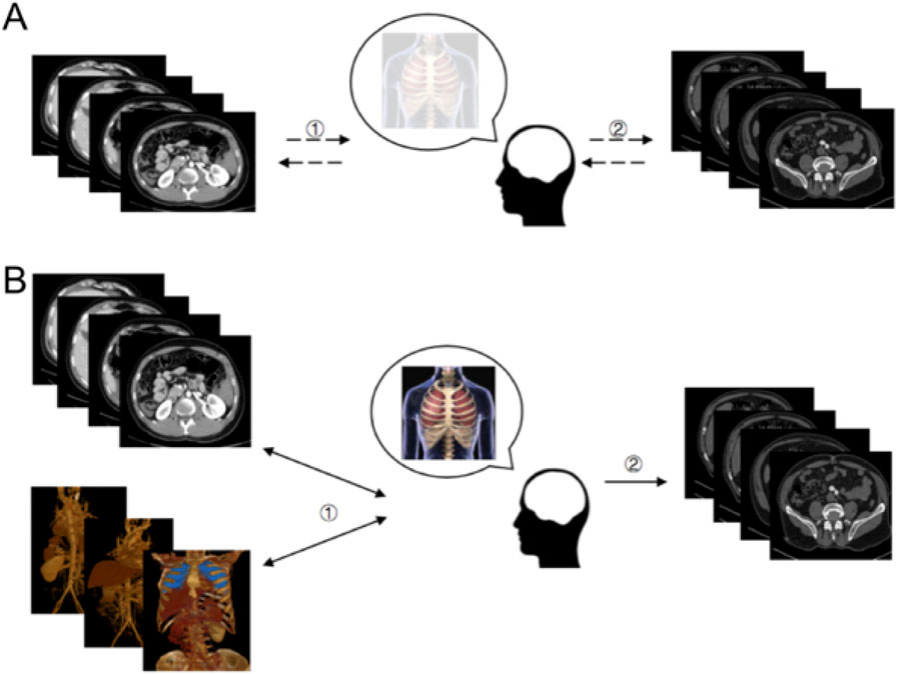
Imaging learning model. (**A**) Classical learning model: “*2D→3D→2D*”. ① Long and difficult learning process that results in vague 3D structures due to lack of immediate correction. ② Process of clinical practice in which residents must correct and rebuild this structure. (**B**) New learning model combining 2D and 3D images: “*2D→3D→3D→2D*”. ① Accelerated learning process in which structures can be corrected in both a timely and repeated manner, which results in accurate 3D structures. ② Process of clinical practice in which the remembered 3D structures are successfully applied.

## R10 Validation of two new simulators of neuraxial anaesthesia: students vs anaesthetists

### Sandra Rubio Bernabe, Javier Pueyo Villoslada, Xabier Unamuno Iñurritegui, Marcos Llorente Ortega, Secundino Fernandez Gonzalez, Cristina Honorato Cia

#### University of Navarra, Navarra, Spain

##### **Correspondence:** Cristina Honorato (chonorato@unav.es)

**Ethics statement**

The authors declare that they have followed the guidelines for scientific integrity and professional ethics. The article does not contain any studies with human or animal subjects.

**Introduction & Aims**

During the learning process, an anaesthetist must achieve multiple skills. Spinal lumbar punctures (SLp) are often learnt as an undergraduate, while epidural thoracic puncture (ETp), is more specific of anaesthetists and is learnt during residency training. Simulation is a recognized tool in the learning process of these abilities, but it is difficult to find simulators that mimic real life sensations accurately. For that reason, we developed two prototypes, and the aim of this study is the validation of the simulators for those two techniques.

**Methods**

Prospective observational study with 19 medical students and 20 anaesthetists divided in 4 groups: for the SLp simulator, students were compared against specialists; for the ETp simulator, 8 anaesthetists not familiarized with thoracic epidurals and 9 anaesthetist with enough experience were selected.

The validation methodology (face, content and construct) was used. For face and content validities, quantitate questionnaires with the Likert Score (1-5) were applied. For the construct validity, time and a checklist of the steps to perform a correct puncture were taken into account.

The statistical analysis was performed using nonparametric analyses: U-Mann Whitney. P-values under 0.05 and medias more or equal than 4/5 were considered significant and valid, respectively.

**Results & Discussion**

For the SLp simulator, both groups had similar scores in usefulness. The group formed by anaesthetists didn’t consider it essential for the learning process (*p*<0.05). The simulator scored high in realism, especially in the palpation of anatomical structures (4.6/5) and the fluid outflow (4.5/5). It is also able to discern novices and experts through time (*p*<0.05).

In the case of ETp simulator, the two groups involved found it useful, as well as essential for training. The simulator scored high in consistency with reality for both groups, obtaining high scores simulating the contact of the needle with the bone (4.5/5), the identification of the ligamentum flavum (4.5/5) and the loss of resistance of the epidural space (4.5/5).

Both simulator scored low for reproducing the needle pass through skin and subcutaneous tissue.

To sum up, both simulators have achieved the face, content and construct validities, although they still have room for improvement. This study shows that these simulators can be useful for training due to their high realism.

## R11 Simulation-based cerebral angiography coiling training performance in novices

### Oleksiy Zaika, Mel Boulton, Roy Eagleson, Sandrine de Ribaupierre

#### Western University, London, Ontario, Canada

##### **Correspondence:** Oleksiy Zaika (ozaika@uwo.ca)

**Ethics statement**

The authors declare that all procedures followed were in accordance with the ethical standards of the responsible committee on human experimentation (institutional and national) and with the Helsinki Declaration of 1975 (in its most recently amended version). Informed consent was obtained from all patients/participants included in the study.

**Introduction & Aims**

Endovascular surgical procedures require visuospatial coordination in work spaces with restricted motion and temporally limited imaging. The development of the skills needed for these procedures can be facilitated by 3-D simulator-based training. Commercial simulators have been developed, but there were no rigorous studies or their value for training. A few studies have been done looking at the ability for the ANGIO Mentor to be an effective training tool in diagnostic cerebral angiography, however, this simulator has not been tested thoroughly in its ability to train aneurysm coiling. Coiling is a particularly difficult task to learn for novices as it requires spatial awareness, fine manipulation, and planning to effectively and successfully pack the aneurysm. We hypothesized that we would see a similar training curve when learning to coil as seen in diagnostic angiography using the simulator. We studied the results in the context of procedural performance compared to visuospatial ability.

**Methods**

In this study, 12 novice medical students were given simulation-based diagnostic cerebral angiography training until a procedural plateau in performance, established in our previous work (Zaika et al., 2016). Subsequently, they were trained using video tutorials and written instructions to identify, measure and intervene with cerebral aneurysms using endovascular coils. Over the span of 6 sessions, participants were assessed on their procedural pace, coiling quantity and quality, and perforation rates. Concurrently, their spatial ability was assessed using a mental rotations test (MRT).

**Results & Discussion**

All individuals were able to perform the procedure faster after 6 sessions, reducing their average time from 42 to 24 minutes. Coil success rate improved over from 82% to 88% and coil packing rate remained consistent at 30% throughout testing. High perforation rate seen at the start of the study showed a trend of decreasing over the latter sessions, however, over half of aneurysms were still being perforated by the novice participants. No change in aneurysm coiling quality was found, with a slight decrease in number of parent artery coil protrusions. High MRT individuals were better able to establish necessary tools prior to coiling, however, no other MRT-specific changes were seen. This work identifies the utility of simulation-based cerebral angiography training in identifying the particular difficulties trainees experience in learning procedural skills, including prevention of perforations, proper positioning and success of coil establishment within the aneurysm.

## R12 An exploratory study of experts’ experiences of how to embed a sustainable simulation-based education program and/or centre in a teaching hospital

### Rebecca Szabo^1^, Robert O’Brien^1^, Margaret Bearman^2^

#### ^1^University of Melbourne, Melbourne, Australia; ^2^Deakin University, Geelong, Australia

##### **Correspondence:** Rebecca Szabo (rebecca.szabo@unimelb.edu.au)

**Ethics statement**

The authors declare that all procedures followed were in accordance with the ethical standards of the responsible committee on human experimentation (institutional and national ) and with the Helsinki Declaration of 1975 ( In its most recently amended version). Informed consent was obtained from all participants included in the study.

Ethical approval for the study was obtained through The University of Melbourne Department of Medical Education Human Ethics Advisory Group (HEAG) (project ID 1749545).

**Introduction & Aims**

Simulation is defined as a technique to replace or amplify real experiences with guided experiences that evoke or replicate substantial aspects of the real world in a fully interactive manner. There is extensive research demonstrating efficacy of simulation to teach practical skills, human factor skills and teamwork and emerging evidence to demonstrate that this translates to patient outcomes. Implementation science scholars argue that the introduction of novel practices into established health care organizations requires much effort and needs to be “informed by an assessment of the likely barriers and enablers”.

Aims:

1. to explore experts’ experiences of barriers and enablers to implementation of a sustainable simulation-based education (SBE) program and/or centre in a teaching hospital;

2. to determine how to embed a SBE program and/or centre in a teaching hospital.

**Methods**

The study used a mixed method design with a dominant qualitative component to explore experts’ experiences of how to embed a sustainable SBE program and/or centre in a teaching hospital. Known experts across Australia and North America were recruited. Ten Australian participants and seven North American participants (2 Canadian, 5 American) were included. To enhance the robustness of analysis a sample of three of the semi-structured interviews (one from each country) was chosen to create an analytical framework using thematic analysis to be used to analyse the remainder of the large data set in the next phase of this research.

**Results & Discussion**

The analytical framework created has demonstrated 6 interlinked themes encompassing both barriers and enablers – 1. Engagement of people, 2. Funding challenges, 3. Executive ‘buy-in’, 4. Context plays a key role, 5. Research and 6. Natural evolution of a program (Fig. 1).

Barriers and enablers are intimately intertwined. Faculty development and promotion of SBE are the most important enablers and funding challenges particularly demonstrating ‘worth’ and ‘return on investment’ are the most significant barriers.

Whilst knowledge of barriers and enablers of SBE is important these will always exist particularly in complex organisations with uncertainty. Leadership traits and skills of SBE directors are vital to overcome any existing or future barriers and to magnify enablers. Increasing understanding of the roles of complexity theory, uncertainty and healthcare as well as change management and leadership to further embed SBE warrants both further consideration and research.

Participants were only from three countries - Australia, USA and Canada. SBE experts from Asia, South America, Europe and Israel may provide different perspectives.

**Fig. 1 (abstract R12).**
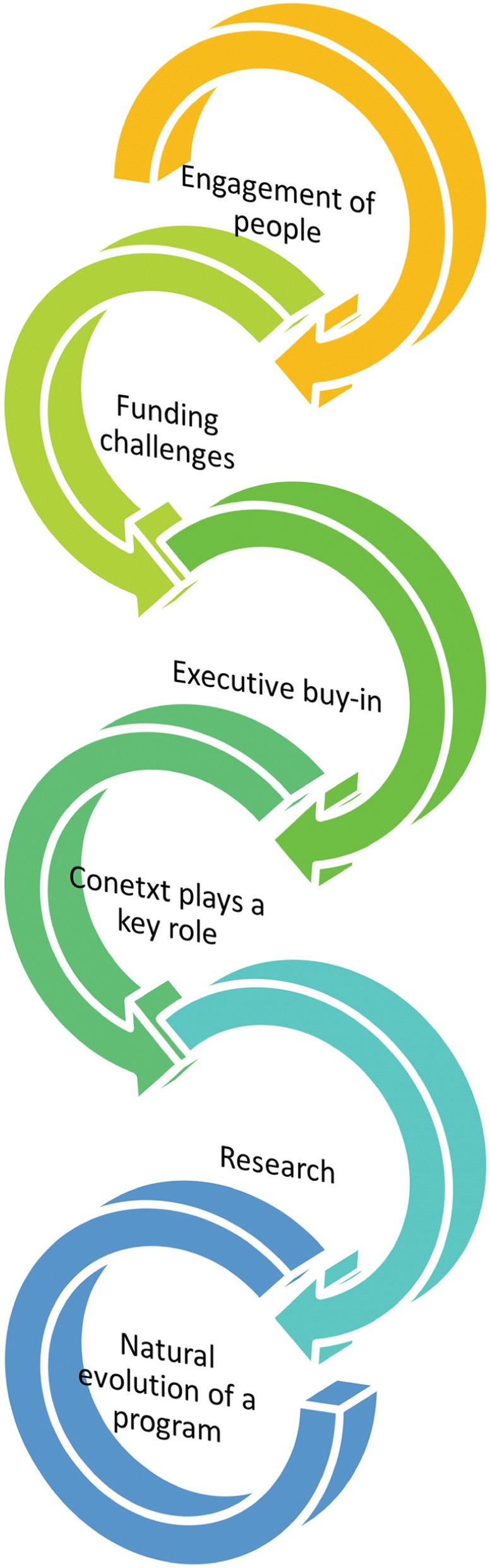
Analytical framework

## R13 High quality CPR - assessment and educational intervention for hospital code teams: a NESERC multicenter simulation based study

### Jesse M. Rideout^1^, Frank L. Overly^2^, Edwin T. Ozawa^3^, Darlene Bourgeois^3^, Micheline Chipman^4^, J. Randy Darby^4^

#### ^1^Tufts Medical Center, Tufts University School of Medicine, Boston, MA, USA; ^2^Rhode Island Hospital, Providence, RI, USA; ^3^Lahey Hospital & Medical Center, Tufts University School of Medicine, Boston, MA, USA; ^4^Maine Medical Center, Portland, OR, USA

##### **Correspondence:** Jesse M. Rideout (rideout@post.harvard.edu)

**Introduction & Aims**

The American Heart Association (AHA) reports only 25% survival rate with in-hospital cardiac arrests (IHCA). Other publications have previously documented the correlation between survival and high-quality CPR (hqCPR). Nevertheless, hqCPR performance: rate 100-120, depth 5-6cm, compression fraction (time in CPR >60%), and peri-shock pauses (<10 sec), varies and research indicates that it is suboptimal despite certification training.

To address this complex issue of technical proficiency and team-work during a code, we developed a 3-hour standardized interprofessional educational intervention, incorporating simulation, didactics, and deliberate practice with a CPR feedback device (ZOLL R-series defibrillator). Our intervention targeted members of hospital code teams at four medical centers, affiliated with the New England Simulation, Education, and Research Consortium (NESERC).

**Methods**

Participant inclusion criteria required current certification (BLS or ACLS) and membership in their hospital’s code team. We recruited ten groups of four participants from each of the four sites (40 teams and 160 participants). Teams managed simulated codes at baseline and post-intervention that had CPR data recorded (utilizing the ZOLL R-series). Additionally, after training, individuals had 2-3 attempts to score >80% for the composite target rate and depth (hqCPR) over 2 minutes. We utilized the paired t-test to assess changes from pre- to post-intervention.

**Results & Discussion**

We analyzed data on 152 individuals across four sites: 43% physicians, 26% nurses, 16% CCTs, 6% RTs, 9% other. Demographics included: median age 29.5; median 2 years on code team; 75% completed AHA certification <12mo; 47% observed CPR during an IHCA <1mo; 37% had performed CPR in >20 IHCA (>50% were involved in >10); 84% rated themselves as confident to extremely confident to perform hqCPR. 97% did not use CPR feedback-devices during hospital codes.

We present data on pre- and post-intervention mean(SD) in Table 1.

Participants in this study were all hospital code team experienced, AHA certified, and very confident in their hqCPR abilities. Individually, their baseline hqCPR score for 2 minutes was only 11.9%. Explanations for such low baseline performance in qualified participants include: challenges of combining target depth and rate simultaneously for 2 min; overconfidence; and underestimating the difficulty of hqCPR. After our brief intervention, we found statistically significant improvements with individual hqCPR (+42.8%) and team hqCPR (+21.7%). Teams also made statistically significant improvements in compression fraction (+5.8%) and peri-shock pause (-5.6s).

**Table 1 (abstract R13).** Baseline and post-training hqCPR means(SD) and differences for code teams and individuals

	n	Pre–Intervention	Post–Intervention	Difference	*p* value
Teams					
hqCPR (%)	37	9.1 (15.2)	30.8 (18.6)	21.73 (19.6)	<0.0001
Rate (%)	37	20.7 (25.8)	67.6 (25.3)	47.0 (29.9)	<0.0001
Depth (%)	37	29.0 (23.3)	42.8 (21.7)	13.8 (20.9)	0.0003
Comp. Fraction %	37	75.7 (25.0)	81.4 (23.5)	5.8 (15.9)	0.03
Perishock Pause (s)	33	15.5 (10.1)	9.9 (6.4)	- 5.57 (7.6)	0.0002
Individuals					
hqCPR (%)	148	11.9 (23.4)	54.7 (31.9)	42.8 (37.3)	<0.0001
Rate (%)	148	29.9 (37.1)	79.9 (26.8)	50.1 (43.9)	<0.0001
Depth (%)	147	32.0 (28.6)	65.1 (29.8)	33.0 (34.6)	<0.0001

## R14 Non-Technical Skills checklist in Postpartum hemorrhage (PPH)

### Job Anais, Michelet Daphné, Barré Jessy

#### Ilumens laboratory, Paris Descartes University, Paris, France

##### **Correspondence:** Michelet Daphné (daphnemichelet@gmail.com)

**Ethics statement**

The authors declare that they have followed the guidelines for scientific integrity and professional ethics. The article does not contain any studies with human or animal subjects.

**Introduction & Aims**

Postpartum Haemorrhage (PPH) is the leading cause of maternal death in the world. Around 100 000 women die each year because of PPH (AbouZahr, 2003). PPH can be considered as a critical situation that generate errors then accidents (St Pierre, Hofinger & Buerschaper, 2008). Beyond impact of the lack of technical competences, some author highlights the role of Non-Technical Skills (NTS) in medical errors (Donaldson, Corrigan, & Kohn, 2000; Makary & Daniel, 2016). Considered as a combination of cognitive, social knowledge and personal resources that complement medical skills, they contribute to an efficient and safe performance in medical activity.

**Methods**

We created a modeling of NTS including behavioral and cognitive markers in PPH situation. From a literature review of NTS in the medical field, we selected all the items that can be relevant in this situation. This first list was completed by video analysis of training sessions using High-Fidelity simulation to validate the relevance of each item. Finally, a questionnaire was submitted to experts in simulation (midwives, obstetricians, anesthetists and maternity nurses) in order to validate our model. The purpose of this questionnaire was to validate the relevance of behavioral markers and mental processes that we previously identified.

**Results & Discussion**

Our exhaustive list of NTS mobilized during PPH, developed from the literature review of the existing tool and completed by video analysis, has highlighted 114 items. All items were included in a questionnaire. Experts evaluated each behavioral and cognitive marker on a 4 point Likert scale, from absent /Not important (1) to Very important (4). Our result show in the questionnaire that 69 items are evaluated as very Important (>3,5), 36 items are evaluated as important (between 3 and 3,5), and 9 items are evaluated as less important (<3). The Content Validity Index (CVI) is used to identify the relevance of our behavioral and cognitive markers : 109 items (/114) had a CVI of greater than 0,75 (acceptable). The others were modified or deleted. We finally kept 111 items in 6 categories: Situation Awareness/Decision Making/Team working/Leadership/Communication and Managing Emotions.

Some studies reveal that 80% of PPH deaths in France can be avoided (Bouvier-Colle, 2007). We present a tool (checklist), to evaluate NTS of midwives, obstetricians, anesthetists, maternity nurses and also the complete team during PPH. Simulation trainers can use this tool to help practitioners to improve their NTS in this critical situation. Next step, is to test reliability and validity of our tool.

**Table 1 (abstract R14).** Extract of the checklist “Non-Technical Skills in Post Partum Hemorrhage”

Non Technical Skill	Example of behavioral or cognitive marker
*Communication*	Keep the husband informed of the situation Targeted communication
*Situation awareness*	Know how to identify clear signals (blood loss volume) React according to collected information
*Decision making*	Quick reaction to diagnosis Events anticipation
*Team working*	Doubts or misunderstandings are verbalized Mutual help between team members
*Leadership/Followership*	The leader delegate tasks Have a dynamic participation
*Emotion management*	Stay focus/ alert Be tolerant with others

## R15 Catch the Ball and Close the Loop: a simulation icebreaker to improve team communication

### Matthew Aldridge, Jeremy Purdell-Lewis, Katherine Finucane

#### Department of Postgraduate Medical Education, North Bristol NHS Trust, Bristol, UK

##### **Correspondence:** Matthew Aldridge (m.j.aldridge@gmail.com)

**Ethics statement**

The authors declare that they have followed the guidelines for scientific integrity and professional ethics. According to the policy activities that constitute research at North Bristol NHS Trust, this work met criteria for operational improvement activities exempt from ethics review.

**Introduction & Aims**

Effective team working and communication has been shown to be an important aspect of patient safety [1]. One example of good communication practice is the use of ‘Closed-Loop Communication’ [2]. This consists of three components; the call-out, check-back, and closed-loop [2]. Increased use of this technique has been associated with higher team performance [3, 4] however knowledge of this technique alone does not translate directly to increased use in practice [5].

This quality improvement project aims to increase the frequency of closed-loop communication during simulation scenarios for junior doctors at North Bristol NHS Trust (Bristol, UK). These sessions consist of a briefing and icebreaker activity followed by simulated medical emergency scenarios, each with a debriefing discussion. The existing icebreaker was adapted to demonstrate closed-loop communication, and the use of this technique in subsequent scenarios was assessed.

**Methods**

During four sessions in October 2017 this icebreaker consisted of a ‘catch-the-ball’ activity; participants were asked to catch a tennis ball, read out a word written on it, and then throw it to another participant (Catch-The-Ball Only [CTBO]). In the third and fourth sessions, participants were asked to repeat this activity using closed loop communication; participants were asked to designate a person, receive a response, and close the loop before throwing the ball (Catch-The-Ball with Closed Loop [CTBCL]).

During the simulation scenarios a faculty member recorded all instances of participant-to-participant or participant-to-confederate information or action requests. These instances were categorised as call-out, call-out with response, or closed-loop communication (CLC).

**Results & Discussion**

We observed 263 instances of participant initiated communication across 10 simulated medical emergencies involving 19 junior doctors. The 5 scripted scenarios used in the first two sessions were identical to those used in the second two sessions.

In the two sessions using the CTBO icebreaker, we observed 118 instances of participant initiated communication of which 20 (17%) used CLC. There was a significant (*p*<0.001) increase in the use of CLC following the CTBCL icebreaker (145 instances, 68 [47%] CLC).

We are encouraged by these results, and will continue to use this modified icebreaker in simulation sessions to improve the quality of team communication. This appears to be an effective learning tool and may help bridge the gap between knowledge of effective communication techniques and establishing their use in practice.

**References**

1. M Leonard, S Graham, D Bonacum. *The human factor: the critical importance of effective teamwork and communication in providing safe care*. Qual Saf Health Care. 2004 Oct; 13(Suppl 1): i85–i90.

2. CS Burke**,** E Salas**,** K Wilson-Donnelly**,** H Priest. *How to turn a team of experts into an expert medical team: guidance from the aviation and military communities.* Qual Saf Health Care. 2004 Oct;13 Suppl 1:i96-104.

3. CA Bowers, F Jentsch, E Salas, et al. *Analyzing communication sequences for team training needs assessment*. Hum Factors. 1998 Dec;40:672–679.

4. D Siassakos, K Bristowe, TJ Draycott, et al*. Clinical efficiency in a simulated emergency and relationship to team behaviours: a multisite cross-sectional study*. Br J Obstet Gynaecol. 2011;118:596–607.

5. Maria Härgestam, Marie Lindkvist , Christine Brulin, Maritha Jacobsson, Magnus Hultin. *Communication in interdisciplinary teams: exploring closed-loop communication during in situ trauma team training*. BMJ Open 2013;3:e003525.

## R16 Decision-making in nursing: how undergraduate students see their learning in high-fidelity simulation

### Cristina Lavareda Baixinho, Helga Rafael Henriques, Cristina Saraiva, Isilda Rebelo, Helena Presado, Sónia Colaço, Isabel Félix

#### Lisbon Nursing School, Lisbon, Portugal

##### **Correspondence:** Cristina Lavaredo Baixinho (crbaixinho@esel.pt)

**Ethics statement**

As authors we declare that we have followed the guidelines for scientific integrity and professional ethics. All participants were informed of the study’s objectives. They accepted to participate and authorized their participation by filling out the informed consent term. Confidentiality of data and anonymity of participants were ensured.

**Introduction & Aims**

Clinical decision-making in nursing is complex. It involves critical integration of evidence, the patient’s preference, the experience, the context, and the available resources among others. There is a demand in health care settings for nurses to use responsibility, autonomy and critical judgement in decision making. The aim of this research is to evaluate the student’s self-perception about decision-making competences developed during high-fidelity simulation practice (HFSP).

**Methods**

This quantitative, cross-sectorial and descriptive study aims to answer the research question: “What is HFSP’s contribution to decision-making learning in nursing undergraduate students?”

The subscale “Decision-Making in Nursing”, from the Scale to “Evaluate the Contributions of High-Fidelity Simulation Practice in Learning the Nursing Process’ Steps”, was used. This subscale has nine (9) items scored on a Likert scale with four (4) options. The questionnaire was sent by Google Drive® to a sample of students who met the inclusion criteria: attend the 5th semester, during clinical practice in medical or surgical wards. Participation consent was requested and data anonymity confidentiality were assured.

**Results & Discussion**

The sample of 135 students answered the questionnaire. The majority, 90.3%, are female; mean age is 20.8 years (standard deviation of 2.72). With internal consistency alpha-Cronbach 0.916 for the nine (9) items, the instrument shows very good internal consistency, a single factor explains 60,119 of the variance. Pearson’s correlation coefficient values, relating each item with the scale total without the item, ranged between 0.752 and 0.825.

In the descriptive analysis it was verified that the students evaluated positively HFSP contribution to decision-making, since in average the subscale scores 30.47 ± 4.47 in 36 possible points. The indicators of the subscale: mobilizing knowledge, structuring clinical reasoning, improving clinical thinking and developing decision-making stand out with a median of 4.

HFSP contributes to the clinical decision-making of the undergraduate students. Future studies should explore methods and techniques that can measure the impact of the simulation in the decision- making in nursing.

## D1 Innovation in patient safety: realistic clinical simulation to design future safer work spaces

### José M Quintillá^1^, Carmen de la Gala^1^, Martí Iriondo^2^, María J Troyano^2^, Carlos Aláez^1^, Gregory Loan^3^, Peter Weinstock^3^

#### ^1^Simulation Program, Hospital Sant Joan de Déu, Barcelona, Spain; ^2^Neonatology Unit, Hospital Sant Joan de Déu, Barcelona, Spain; ^3^Boston Children’s Hospital Simulator Program, Boston, MA, USA

##### **Correspondence:** José M Quintillá (joseqm@hsjdbcn.org)

**Ethics statement**

The authors declare that they have followed the guidelines for scientific integrity and professional ethics. The article does not contain any studies with human or animal subjects.

**Introduction & Aims**

The Agency for Healthcare Research and Quality defines “high reliability organizations” as organizations that operate in complex, high-hazard domains for extended periods of time without serious accidents or catastrophic failures. It assigns 5 characteristics to this type of organizations: preoccupation with failure, reluctance to simplify, sensitivity to operations, deference to expertise and commitment to resilience. In this mental framework of culture for safety, our Simulation Program has a specific service line that uses simulation as a test tool to help design spaces and ways of working safer and with a better experience of professionals and families

The aim of this study was to analyze, through a highly realistic clinical simulation, the design of the future Neonatal Intensive Care Unit (NICU), still to be built, in relation to the minimum single box size.

**Description**

The activity was designed and executed jointly between the Sant Joan de Déu Simulation Program and the Boston Children’s Hospital Simulator Program. A working group was created, consisting of health care professionals, experts in clinical simulation and engineering professionals.

In the preparation phase a main objective was identified (to study if it was possible and safe to perform complex procedures in a single box of 15, 18 or 21 m2) and a secondary objective (to identify critical elements in the layout of the equipment and in the dynamics of work in relation to spaces).

Using specific high fidelity mannequins, 3 scenarios were executed: stabilization of a newborn with advanced life support, entry in ECMO (jugular cannulation and circuit and pump connection) and open abdominal surgery within the NICU. The scenarios were tested in the different sizes of box proposed. The event lasted 2 days and 38 people took part, including the organizers, the technical team, the clinical participants and the observers.

Relevant conclusions were reached about the minimum size of box (resuscitation was possible in 15 m2, surgery in 18 m2 and ECMO in 21 m2) and necessary determinants were identified in terms of disposition of equipment and work dynamics to maintain safety.

**Discussion**

Highly realistic clinical simulation is a very useful methodology to help design safer workspaces. It allows the participation of professionals of different profiles, who share their visions based not on predefined ideas, but on experiences lived through simulation.

## D2 Grappling with Evaluation - analysis of the impact of a Simulation Train-the-Trainer course

### Huon Snelgrove, Jasmine Burnett, Amber Savary-Trathan, Christopher Broom, Argyro Zoumprouli, Asanga Fernando

#### St George’s University Hospitals NHS Foundation Trust, London, UK

##### **Correspondence:** Huon Snelgrove (snelgrovehuon@me.com)

**Ethics statement**

Informed consent was obtained from all participants included in the study. All institutional and national guidelines for the care and use of laboratory animals were followed.

**Introduction & Aims**

As an educational strategy clinical simulation in hospitals provides the opportunity for learning that is both immersive and experiential. Thus to improve education and ultimately enhance patient safety, clinical teachers are attending Train-the Trainer courses to expand their repertoire of teaching skill so that they can include simulated patients, mental simulation, manikin simulators and task trainers into their teaching.

We aimed to evaluate the impact of a two-day clinical simulation Train-the Trainer courses on 50 clinical teachers (including nurses and doctors) over a 12 month period following their training.

We looked at impact in terms of frequency, modality and teachers’ theories of use. We also identified barriers and facilitators that influenced whether participants were able to embed simulation in their clinical contexts.

**Description**

Using mixed methods our data gathering included:

1. A post course evaluation survey delivered to participants at between 3-12 months following their training.
2. Two focus groups with participants.
3. Simulation centre data over 12 months on frequency and type of simulator borrowings for mobile in situ simulations in the hospital.

**Discussion**

The Train-the-Trainer course provides exposure to a mix of simulation modalities and theories of use but how this impacts on clinical teaching practice following training is often difficult to gauge. In our 12 month follow up we identified a number of impacts including barriers and facilitators for the adoption by clinical teachers of simulation in clinical areas. Time and logistical barriers created special challenges for front line clinical teachers who want to adopt simulation-based learning.

The use of ‘mental rehearsal’ or ‘mental simulation’ was the most easily transferable outcome from the Trainer course. Course. Participant composition had an important effect on impact. Junior doctors reported fewer opportunities and more variable experiences in using simulators. In contrast, practice nurse educators represented a solid group of end users and were more likely to adopt and embed simulation - part task and simulator - in in situ training. In cash strapped hospital systems this may suggest a strategic case for identifying professional groups, in the hospital who have front line teaching responsibilities for preferential inclusion in Train-the-Trainer courses.

## D3 Enhancing perceptions of Paediatrics and promoting speciality recruitment using high fidelity simulation

### Peter Mallett^1^, Carol Junk^2^, Thomas Bourke^2,3^, Andrew Thompson^2^

#### ^1^Department of Paediatric Simulation & Education, Royal Belfast Hospital for Sick Children, Belfast, UK; ^2^Department of Paediatrics, Royal Belfast Hospital for Sick Children, Belfast, UK; ^3^Department of Medical Education, Centre for Medical Education, Queen’s University Belfast, Belfast, UK

##### **Correspondence:** Peter Mallett (peter.mallett@belfasttrust.hscni.net)

**Ethics statement**

The authors declare that they have followed the guidelines for scientific integrity and professional ethics. The article does not contain any studies with human or animal subjects.

**Introduction & Aims**

Paediatrics, like many specialties, is experiencing a decline in applications for specialty training [1]. Reasons include perceptions of poor flexibility; arduous training programme and lack of adequate career guidance and support [2]. The Royal College of Paediatrics and Child Health (RCPCH) suggest strategies to increase recruitment should include exposure to educational opportunities [2]. In the UK, the transition between foundation level training and specialty training is an uncertain and stressful time [3]. We believe that allowing access to high fidelity simulation training affords a unique opportunity to showcase our specialty.

**Description**

We designed, delivered and evaluated ‘A Foundation in Acute Paediatrics Simulation’ (FAPS) course aimed at offering junior doctors an introduction into the management of common paediatric conditions. A highly experienced inter-professional faculty provides an insight into a career in paediatrics, their own career perspectives and an opportunity for group discussion and tailored personal career advice. Clinically relevant interactive simulation scenarios offer the candidates the opportunity to work alongside colleagues and encounter common paediatric conditions, potentially developing their clinical acumen and enhancing non-technical skills such as teamwork and communication.

16 candidates took part in the pilot FAPS course. Prior to the course 11/16 (69%) candidates were unsure whether they were going to apply for paediatrics. After the course all 11 candidates indicated that that they were more likely to apply [mean score 2.9 before Vs 4.0 after; 1-very unlikely, 3-undecided, 5-Very likely to apply]. 15/16 candidates (94%) felt more confident in the assessment of the unwell child following the course, and all candidates (100%) would recommend this course to peers. Qualitative comments included ‘Excellent concept, relevant scenarios and useful course. Thoroughly enjoyable.’

**Discussion**

This is the first use of high fidelity simulation to enhance specialty recruitment that we are aware of. This course affords an opportunity to gain access to motivated clinicians while experiencing common paediatric conditions in a safe, simulated learning environment. The tailored career advice may be of use for their future speciality direction. This course actively helps in addressing the current plight of low trainee recruitment and retention in Paediatrics and could be easily replicated in other areas.

**References**

1. Royal College of Paediatrics and Child Health. ST1 Paediatric Deanery/LETB Competition ratios and fill rate 2016. London, 2016. http://www.rcpch.ac.uk/.

2. Jacob H, Shanmugalingam S and Kingdon C. Recruitment and retention in paediatrics: . Arch Dis Child. December 2016. doi:10.1136/archdischild-2016-311390.

3. Pinnock R, Reed P, Wright M. The learning environment of paediatric trainees in New Zealand. J Paediatr Child Health 2009;45:529–34.

## D4 Evaluation of the Efficacy of training in cardiac auscultation in medical students at the Agostinho Neto University, Faculty of Medicine, Angola, 2017

### Rosalina Lufefena Nunes^1^, Agostinho Napato^2^, Ricardo Bunda^2^, Mário Santos^2^, Sílvia Lutucuta^2^, José Lopes Martins^2^, Emanuel Catumbela^2^, Maria Fernanda Dias^2^

#### ^1^Faculty of Medicine, Agostinho Neto University, Luanda, Angola; ^2^Department of Medicine, Faculty of Medicine, Agostinho Neto University, Luanda, Angola

##### **Correspondence:** Emanuel Catumbela (ecassoco@gmail.com)

**Ethics statement**

The authors declare that all procedures followed were in accordance with the ethical standards of the responsible committee on human experimentation (institutional and national ) and with the Helsinki Declaration of 1975 ( In its most recently amended version ). Informed consent was obtained from all participants included in the study.

**Introduction & Aims**

Medical simulation is an important tool in the teaching and learning process in medicine, offering students knowledge for the management of well-being, discarding in this phase of learning the potential risks to the patient and the student, giving possibility of repetition from exercise to gaining skills and competence without the handling of patients. The aim of this study was to evaluate the efficacy of cardiac auscultation learning by simulation, comparing it to the regular / traditional teaching of the 3rd year students of the Agostinho Neto University School of Medicine (FMUAN) in the academic year 2017/2018.

**Description**

A controlled clinical trial of educational intervention was carried out with 117 students, divided into two groups: group A 59 (control) and group B 58 (case) and both were submitted to a pre-test in simulators. Group A had traditional classes (in the ward) and group B classes in simulators (laboratory) on cardiac auscultation. All groups had 6 hours of training divided into 2 hours per day on 3 consecutive days. Real patients - with physiological and pathological sounds were randomly selected in the cardiology ward by the team of instructors. For training and testing with simulators were used mannequins of cardiac auscultation type NASCO auscultation trainer and SmartScope, which generates 12 different sounds, namely: normal heart, holosystolic blows, protosystolic, mesosystolic, holodiastolic, protodiastolic, mesodiastolic S3 and S4 gallops, systolic clicks , atrial septal defect, patent ductus arteriosus, ventricular septal defect, on the anterior aspect of the thorax. After the classes were submitted to a test in real patients. Data processing was done through SPSS 23 edition. To compare learning effectiveness, the chi-square test and the McNamar test with a significance level of 0.05 were used.

**Discussion**

Correct responses in cardiac auscultation assessment in real patients between group A and group B after training averaged for group A was 2.15 ± 0.84 and for group B was 3.16 ± 0.62 with statistical significance (*P*<0.05).

Group B had better ability in cardiac auscultation and ability to differentiate between normal and pathological sounds compared to group A. Simulation teaching should be incorporated into the formal teaching process in FMUAN.

## D5 Training healthcare teams with help from the arts

### Anna Jones^1^, Gabriel Reedy^1^, Peter Jaye^2^, Colette Laws-Chapman^2^

#### ^1^King’s College London, London, UK; ^2^Guy’s and St Thomas’ NHS Foundation Trust, London, UK

##### **Correspondence:** Gabriel Reedy (gabriel.reedy@kcl.ac.uk)

**Ethics statement**

The authors declare that all procedures followed were in accordance with the ethical standards of the responsible committee on human experimentation (institutional and national ) and with the Helsinki Declaration of 1975 ( In its most recently amended version ). Informed consent was obtained from all patients/participants included in the study.

**Introduction & Aims**

An innovative three-year collaboration sought to bring some of the techniques used in the performing arts into a team-based training programme in a simulation setting. The programme created a series of unique arts-based training courses designed to enhance care and compassion in healthcare professionals, focusing on skills like resilience, self-care, teamwork, stress-management, non-verbal communication, decision-making, and care and compassion. This research presents the results of programme evaluation, which gathered extensive qualitative and quantitative data on the courses as they were designed and delivered. The evaluation team also presents data gathered from the evaluation of the process of bringing an arts-based organisation together with a team from a healthcare simulation facility to engage in a collaborative development programme.

**Description**

Participants on the innovative training courses, designed collaboratively by simulation educators and performing artists, completed pre- and post-course surveys designed to measure their self-reported confidence on a number of factors and knowledge measures related to the learning outcomes of the courses. Participants volunteered to be contacted for longitudinal follow-up interviews and were asked about the extent to which learning was transferred to practice, and whether behaviour change related to course learning outcomes had occurred. Educators from both teams (simulation and performing arts) were interviewed extensively about the design and collaboration of the programme throughout its development.

**Discussion**

On self-reported measures, participants rated all aspects of the courses very highly (over 90% on most items). At 3 and 6 months after the course, those participants who agreed to be interviewed were positive about the course and were confident that they had changed their thinking and/or behaviour. In particular these positive behavioural shifts were around communication, in particular non-verbal communication, awareness of others, how to deal with emotions, and physical awareness.

From the interviews with the development team, it is clear that although the development process was difficult at times, simulation faculty reported that they had gained a range of skills and changed the ways in which they conceptualise care and compassion and that this has provided a framework that they already include in courses outside of the project. The intervention of a group with totally different ways of thinking and working had provided a fresh perspective and had caused them to question what they do and think about how to do this differently.

